# Bleeding management in pediatric patients

**DOI:** 10.1186/1824-7288-40-S1-A13

**Published:** 2014-08-11

**Authors:** Paola Giordano, Giuseppe Lassandro

**Affiliations:** 1 Dipartimento di Scienze Biomediche e Oncologia Umana, Clinica Pediatrica F. Vecchio, Università degli Studi di Bari “Aldo Moro”, Bari, Italy

## 

Bleeding has always been an alarming clinical symptom in all human societies, and physicians have had varying degrees of success in diagnosing and treating bleeding patients [1]. Because bleeding is part of the human experience, one the most challenging tasks for a physician is to discriminate between “normal” and “pathologic” bleeding. Hemorrhages or bleeds may occur in every tissue and organ. Every bleeding symptom may, however, vary greatly in terms of magnitude: for instance bleeds in the subcutaneous tissues may present as small pinpoint lesions (petechiae) or large bruises, and epistaxis may range from some blood-streaked mucus to a massive hemorrhage [2]. Minor bleeding is a broad category encompassing a wide variety of symptoms that are, however, severe enough to interfere with the patient’ everyday life. Major bleeding defines those episodes that may cause permanent damage to the patient or threaten his or her life. Major bleeding is defined as bleeding in a critical area (intracranial, intraspinal, intraocular, retroperitoneal, intra-articular or pericardial, or intramuscular with compartment syndrome) resulting in an hemoglobin fall > 2 g/dl or requiring transfusion [3]. Bleeding in a child can be a diagnostic challenge because of the wide range of causes, but making a specific diagnosis is clinically important in order to provide appropriate therapy. Bleeding disorders can be inherited or acquired, and include coagulation factor deficiencies or Von Willebrand diseases, platelet deficiencies and/or dysfunctions, blood vessels alterations [4]. The evaluation of a child presenting with bleeding should include a comprehensive medical and bleeding history, a complete family history, a detailed examination and selected laboratory tests. For immune thrombocytopenia, in newly diagnoses children, intravenous human immunoglobulin or corticosteroids can be used [5]. Individuals with platelet function defects should be managed by qualified specialist and platelet inhibitor medication should be avoid. First line drugs for patients with platelet dysfunction are: antifibrinolytics, desmopressin, platelet transfusions and recombinant factor VIIa [6,7]. Bleeding events still occurs in patients with hemophilia despite prophylactic factor replacement. Adequate management depends not only upon the hemophilia subtype and site of bleeding, but also the episode severity, identifiable precipitants (e.g. trauma), the patients’ age and current place of residence, adequacy of venous access, presence of inhibitors, previous history of bleeding, and the time of onset in relation to prophylactic therapy. For all diseases other important initial considerations include the applicability of first aid measures (e.g ice, direct pressure, splinting) and appropriate analgesia [8].
